# TIM-3 and TIM-1 Could Regulate Decidual *γδ*TCR Bright T Cells during Murine Pregnancy

**DOI:** 10.1155/2019/3836942

**Published:** 2019-05-20

**Authors:** Jasper Nörenberg, Matyas Meggyes, Pal Jakso, Eva Miko, Aliz Barakonyi

**Affiliations:** ^1^Department of Medical Microbiology and Immunology, Medical School, University of Pecs, Pecs, Hungary; ^2^Janos Szentagothai Research Centre, Pecs, Hungary; ^3^Department of Pathology, Medical School, University of Pecs, Pecs, Hungary

## Abstract

Pregnancy is an immunological enigma where paternal antigens are present at the fetomaternal interface. What regulates the local immunotolerance, which is necessary to prevent rejection of the conceptus, is still under strong investigation. Gamma/delta T cells are believed to play a role in the local regulation of this immunotolerance towards the semiallogenic fetus. Gamma/delta T cells from the uterus and spleen of pregnant and nonpregnant mice were analyzed by flow cytometry. We confirmed that the rate of *γδ*T cells in the decidua increases during murine pregnancy and half of decidual *γδ*T cells are CD4+. Furthermore, we found a unique association of CD4 or CD8 coreceptor expression with their *γδ*TCR intensity, where in all investigated groups CD4- or CD8-positive *γδ*T cells seemed principally to be *γδ*TCRdim. In addition, compared to peripheral *γδ*T lymphocytes, a greater proportion of decidual *γδ*T cells expressed the cytotoxic marker CD107a and markers of Th1 or Th2 polarization (TIM-3, TIM-1), where decidual *γδ*TCRbright cells were characterized by high TIM-3 and TIM-1 receptor expression. On the other hand, no difference in the expression of CD160, a receptor with dual function affecting cytotoxicity and T cell inhibition, was detected. Within lymphocytes expressing CD107a, TIM-1, or CD160, the rate of *γδ*T cells was significantly higher in the decidua. According to our results, cytotoxic potential of decidual *γδ*TCRbright cells could be regulated by TIM-3 ligation, while the TIM-1 receptor seems to be able to influence the Th1-Th2 balance at the fetomaternal interface. These mechanisms could play a part in the active maternal immunotolerance towards the fetus, allowing an efficient protection against pathogens during healthy murine pregnancy.

## 1. Introduction

In pregnancy, a distinct balance of T helper- (Th-) mediated immunity is crucial to provide protection against pathogens, while mediating tolerance towards the semiallograft [1–3]. It is still unclear how this balance is adjusted exactly. T cell immunoglobulin domain and mucin domain-containing molecule- (TIM-) 3 is expressed on Th1 cells [4]. Engaging galectin-9 (Gal-9) probably modulates the balance of Th1 and Th2 cytokines, by downregulating the Th1 response, which is crucial for allograft tolerance [5, 6]. Together with programmed cell death protein- (PD-) 1 and other inhibitory receptors, TIM-3 mediates CD8+ T cell exhaustion [7]. TIM-3 can be found on activated human natural killer (NK) cells, where it suppresses the NK cytotoxicity [8]. Over 60% of decidual NK cells are TIM-3+ and produce high levels of Th2 cytokines [9], which seem to be essential for a successful pregnancy [10]. In contrast, TIM-1, which is a receptor for phosphatidylserine and TIM-4, is mainly expressed on Th2 cells and has an activating function [11–14]. However, the results of this activation appear to be different according to the type of ligation [12]. Xiao et al. described that proinflammatory as well as antiinflammatory cytokine production can be triggered by distinct types of anti-TIM-1 monoclonal antibodies [15]. In addition, it has been also shown that the engagement of agonistic anti-TIM-1 monoclonal antibodies in CD4+/forkhead box protein 3 (FOXP3)+ cells leads to downregulation of FOXP3 [16], which is a commonly used marker for regulatory T cells [17]. Despite the evidences about the influence of TIM-1 in allograft tolerance [18], the presence and role of TIM-1 receptor at the fetomaternal interface are poorly investigated.

Immune cells must recognize maternal and paternal antigens of the trophoblast, which leads to the regulation of Th1-Th2 cytokine balance. CD160 could participate in this immunological signaling, because it is capable of binding to classical and nonclassical major histocompatibility complex (MHC) class I molecules, like human leukocyte antigen- (HLA-) A, HLA-B, HLA-C, HLA-E, and HLA-G [19–21]. Beside *γδ*T cells, all intestinal intraepithelial T lymphocytes and some *γδ*T cells and CD56dim NK cells express CD160 [19, 22–24], which is a marker for cytotoxicity on NK cells [19] and could also enhance NK cell-derived Th1 cytokine production [21]. According to our present knowledge, herpes virus entry mediator (HVEM) is a second ligand for CD160, which could deliver cell survival promoting inhibitory signaling [24–26]. Similarly to HLA-C, HLA-E, and HLA-G molecules, HVEM is also expressed in the placenta [27–30]. Therefore, CD160 could be able to regulate cell survival and function during implantation or maternal tolerance.

Gamma/delta T cells, in general, have been the scope of intense investigations over the last decades. They are able to react on a MHC-non-restricted manner and are strong in cytotoxic potential and cytokine production, and they expand during pregnancy [31]. Thus, *γδ*T cells presumably play an important role in shaping the pro-/anti-inflammatory balance of the fetomaternal interface. Indeed, human decidual *γδ*TCR+/CD56dim cells show TGF*β* and IL-10 immunosuppressive cytokine profile on the mRNA level [32]. Afterwards, Fan et al. did confirm that around half of decidual *γδ*T cells release these Th2 cytokine proteins and that decidual *γδ*T cell-derived IL-10 enhance human trophoblast proliferation and invasion [33].

However, *γδ*T cells seem to play a significant role in the maintenance of healthy pregnancy; the exact mechanisms are still poorly understood. In this study, we focused on the phenotypical aspects of *γδ*T cells at the fetomaternal interface. Since healthy human decidual samples are hardly available, a mouse model was necessary to acquire in vivo data. Therefore, we implemented a mouse model with the objective of investigating the expression of CD4, CD8, CD107a, TIM-3, TIM-1, and CD160 within *γδ*T cell populations of the uterus and the spleen in nonpregnant and pregnant mice. We aimed to examine the effect of pregnancy on the ratio of positive cells and the cell surface density of the above markers.

## 2. Materials and Methods

### 2.1. Animal Model

In our mouse model, the same experimental setting was used as in our previous work [34], which is described briefly in each chapter below.

Twenty-four pathogen-free BALB/c mice (2 months old) were kept on a 12 h light/dark cycle at 20-22°C and 40-60% humidity and were fed with standard food pellets and tap water. For fertilization, potential mates were paired up every evening and examined for the presence of the copulatory plug in the next morning. As soon as a copulatory plug was detected, it was considered as gestation day 0.5. Gestating female mice (*n* = 14) were killed on gestation day 14.5 by cervical dislocation, and the spleen and the uterine horns were harvested aseptically afterwards. Nonpregnant female mice (*n* = 10) were used in this experiment as well and were killed at the age of 3 months. The mice were purchased from Pécs Experimental Central Animal Laboratory. Animal housing, care, and application of experimental procedures were in accordance with institutional guidelines under approved protocols (No. BA02/ 2000-6/2012, National Food Chain Safety and Animal Health Control Office of the Government Office of County Baranya). Concerning animal welfare, all efforts were made to minimize suffering.

### 2.2. Isolation of Mononuclear Cells from the Decidua

Following our protocol, described previously [34], after the mice were killed, the abdomen was carefully opened and access to the uterus was gained by pushing intestinal tissue to the side. The uterus was then removed by surgical cuts at the cervix and the ovaries. Then, the uteri were fixed to a clamp at the cervix, which gave enough stability and allowed carefully cutting along the uterine horns. Then, the decidua was separated from the placenta disc under a dissecting microscope. The average number of deciduae per mouse was 5.5. Isolated deciduae were pooled, sliced with scissors, and digested with type IV collagenase (Sigma-Aldrich) at 37°C for 30 minutes. Thereafter, the isolated cells were collected in a fresh tube through a 70 *μ*m nylon cell strainer (BD Biosciences). Subsequently, cells were washed in RPMI 1640 medium (Lonza) supplemented with penicillin (1 × 10^5^ U/l, Lonza) and streptomycin (0.05 g/l, Lonza). The supernatant was aspirated, and the pellet was resuspended in PBS and filtered via a 40 *μ*m nylon cell strainer (BD Biosciences). Finally, the isolated cells were resuspended in RPMI 1640+10% fetal bovine serum (FBS) (Gibco).

### 2.3. Isolation of Mononuclear Cells from the Endometrium

Endometrial mononuclear cells from the nonpregnant endometrium were isolated as described previously [34]. Here, the uterus was removed from the abdominal cavity, sliced with scissors, and digested with type IV collagenase at 37°C for 30 minutes. The isolated cells were collected in a fresh tube, filtered through a 70 *μ*m nylon cell strainer, and washed in RPMI 1640 medium supplemented with penicillin and streptomycin. The supernatant was aspirated, and the pellet was resuspended in PBS and filtered via a 40 *μ*m nylon cell strainer. Finally, the isolated cells were resuspended in RPMI 1640+10% FBS.

### 2.4. Isolation of Mononuclear Cells from the Spleen

According to our previous protocol [34], the spleens were cut into small pieces by a sharp sterile surgical knife, immersed in 2 ml PBS, and pushed gently through a 70 *μ*m nylon cell strainer with a syringe plunger. After that, cells were washed in PBS. Supernatant was aspirated, and the pellet was resuspended in PBS and filtered again via a 40 *μ*m nylon cell strainer. Then, mononuclear cells were separated by Ficoll-Paque gradient (GE Healthcare). Isolated cells were collected and resuspended in RPMI 1640 medium supplemented with penicillin, streptomycin, and 10% FBS.

### 2.5. Fluorochrome Labelling and Flow Cytometric Analysis

The isolated mononuclear cells (10^6^ cells in 100 *μ*l PBS/tube) were incubated with the fluorochrome-labelled monoclonal antibodies at room temperature for 30 min. Hereafter, the cells were washed with PBS and resuspended in a 1% paraformaldehyde (PFA) solution in PBS and stored darkly at 4°C until fluorescence-activated cell sorting (FACS) analysis, performed using a CyFlow® Space flow cytometer (Sysmex Partec GmbH, Görlitz, Germany) equipped with FloMax 2.60 for data acquisition. For analysis, FCS Express 3.0 (De Novo Software, Glendale, CA, USA) was used. The following monoclonal anti-mouse antibodies were used: peridinin-chlorophyll-protein complex- (PerCP-) conjugated anti-mouse CD45 (clone: EM-05; Exbio, Olomouc, Czech Republic), fluorescein isothiocyanate- (FITC-) conjugated anti-mouse CD4 (clone: RM4-5; BD Pharmingen, Franklin Lakes, NJ, USA), FITC-conjugated anti-mouse CD8 (clone: 53-6.7; BD Pharmingen), phycoerythrin- (PE-) conjugated anti-mouse *γδ*TCR (clone: GL3; BD Pharmingen), Alexa Fluor 647- (AF647-) conjugated anti-mouse CD160 (clone: CNX46-3; eBioscience, Waltham, MA, USA), FITC-conjugated anti-mouse CD107a (clone: 1D4B; BD Pharmingen), PE-conjugated anti-mouse TIM-3 (clone: 215008; R&D Systems, Minneapolis, MN, USA), and PE-conjugated anti-mouse TIM-1 (clone: RMT1-4; BioLegend, San Diego, CA, USA). Control antibodies included isotype-matched FITC-, PE-, PerCP- and Alexa Fluor 647-conjugated rat antibodies (all from BD Pharmingen).

### 2.6. Activation for CD107a Functional Assay

The cells were incubated for 4 h (37°C, 5% CO_2_) with the fluorochrome-labelled anti-CD107a antibodies in RPMI 1640 medium, supplemented with penicillin, streptomycin, and 10% FBS; furthermore, ionomycin (1 *μ*g/ml) (Sigma-Aldrich) and phorbol myristate acetate (PMA) (25 ng/ml) (Sigma-Aldrich) were used to activate the cells. Before labelling the cells with the other fluorochrome-labelled monoclonal antibodies, the cells were washed and resuspended in PBS. Finally, cells were fixed in 1% PFA and evaluated by FACS as described in the previous paragraph.

Despite the possibility that the expression of CD160 might change upon activation [35], in our protocol, as described previously, a short stimulation of 4 hours with PMA/ionomycin did not influence the surface expression of CD160 significantly [34].

### 2.7. Statistics

For comparison of data, we performed multiple types of statistical analysis performed using Microsoft Excel version 15.15. We used paired-samples *t*-test to evaluate the relation of two corresponding data from the same mouse and independent-samples *t*-test to compare pregnant and nonpregnant mice. The results presented in the text represent the mean ± SEM of the corresponding set of data. Differences were determined as significant, if the *p* value was equal to or less than 0.05.

## 3. Results

### 3.1. Expression of *γδ*TCR on Lymphocytes in the Uterine and Splenic Samples in Pregnant and Nonpregnant Mice

**Figure 1:**
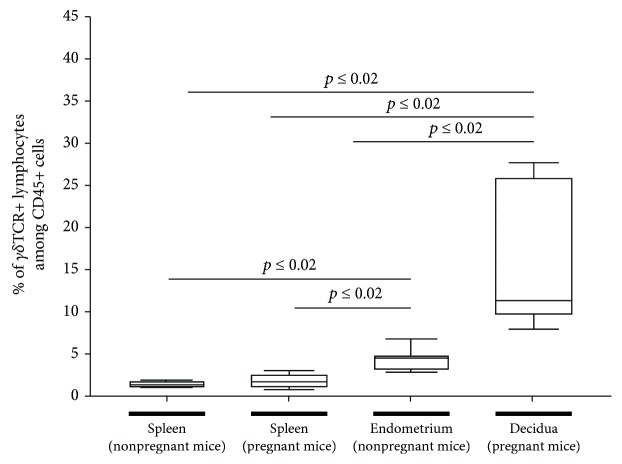
Percentage of *γδ*TCR+ lymphocytes depending on the sample's origin in pregnant and nonpregnant mice. The rate of *γδ*TCR+ cells in pregnant and nonpregnant uterine samples compared to peripheral samples (pregnant and nonpregnant spleen) as depicted by boxplots. The middle line of the boxplots represents the median, the lower box bound the first quartile, and the upper box bound the third quartile. The 95% confidence interval of the mean is represented by whiskers. Each boxplot represents the results of at least six independent experiments.

This study is focused on different subsets of *γδ*T cells in pregnant and nonpregnant mice. The ratio of *γδ*T cells to other lymphocytes depending on the sampled tissue was foremost investigated. Therefore, mononuclear cells, gated on CD45+, of the decidual tissue in pregnant and endometrial tissue in nonpregnant mice were analyzed. Lymphocytes from pregnant and nonpregnant splenic samples were used as control groups. The ratio of *γδ*T cells in uterine samples is significantly higher compared to that in the peripheral control (15.25 ± 0.03 in decidual and 4.88 ± 0.01 in endometrial vs. 2.01 ± 0.00 in pregnant and 1.58 ± 0.00 in nonpregnant splenic samples, *p* ≤ 0.02). Furthermore, decidual lymphocytes express *γδ*TCR significantly more often than lymphocytes of endometrial samples (*p* ≤ 0.02) (Figure 1).

### 3.2. CD4 and CD8 Phenotype of *γδ*T Cells in the Uterine and Splenic Samples in Pregnant and Nonpregnant Mice

**Figure 2:**
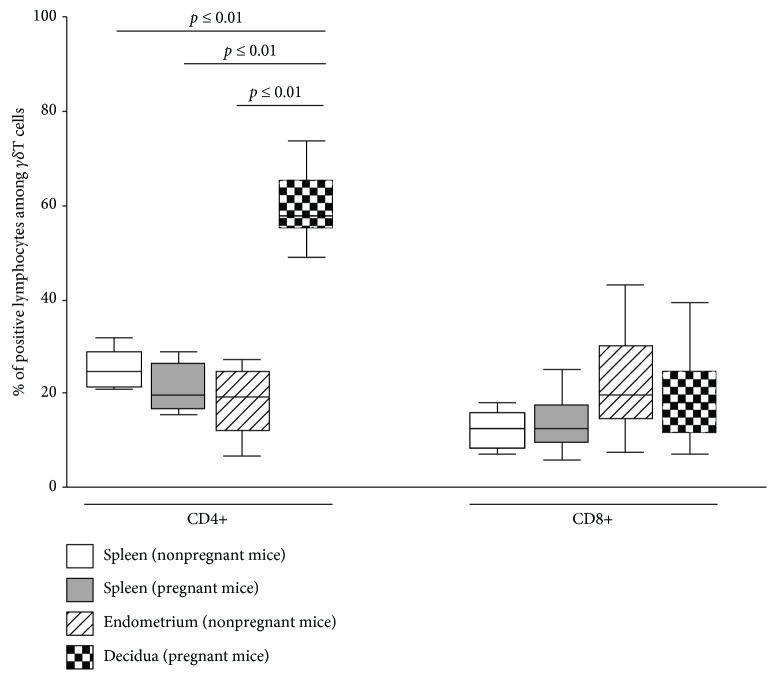
CD4 and CD8 phenotype of *γδ*T cells in pregnant and nonpregnant mice. The rate of CD4+ and CD8+ cells among *γδ*T cells in pregnant and nonpregnant uterine samples compared to peripheral samples (pregnant and nonpregnant spleen) as depicted by boxplots. The middle line of the boxplots represents the median, the lower box bound the first quartile, and the upper box bound the third quartile. The 95% confidence interval of the mean is represented by whiskers. Each boxplot represents the results of at least six independent experiments.

The prevalence of CD4-expressing *γδ*T cells was significantly higher in the decidua than in the other groups, while no difference was observed in the prevalence of CD4+ *γδ*T cells between pregnant splenic, nonpregnant splenic, and endometrial samples (60.28 ± 3.15 in the decidua vs. 18.37 ± 3.19 (*p* ≤ 0.01) in the endometrium vs. 25.51 ± 5.53 (*p* ≤ 0.01) in the pregnant spleen vs. 25.45 ± 1.90 (*p* ≤ 0.01) in the nonpregnant spleen). For CD8 positivity, no significant difference between the four groups was identified (19.04 ± 3.65 in the decidua vs. 22.64 ± 6.23 in the endometrium vs. 13.54 ± 2.17 in the spleen of pregnant mice vs. 12.05 ± 1.82 in the spleen of nonpregnant mice) (Figure 2).

**Figure 3:**
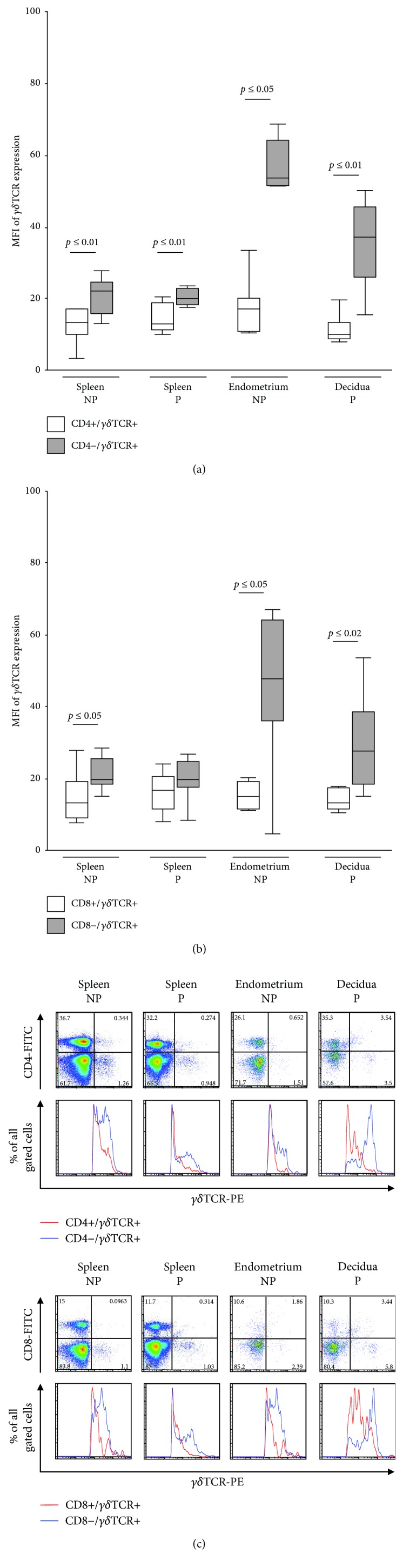
The two *γδ*T cell subsets. Density of *γδ*TCR on CD4- (a) and CD8- (b) positive or negative *γδ*T cells presented as boxplots. The middle line of the boxplots represents the median, the lower box bound the first quartile, and the upper box bound the third quartile. The 95% confidence interval of the mean is represented by whiskers. Each boxplot represents results of at least five independent experiments. (c) Representative dotplots and histograms of all sampled tissues, gated on CD45+ cells. Gamma/delta TCR-PE positivity is represented by the logarithmic *x*-axis. CD4-FITC and CD8-FITC positivity is shown on the respective logarithmic *y*-axis. Related histograms presenting CD4+ or CD4- (CD8+ or CD8-, respectively) *γδ*T cells. Gamma/delta TCR-PE positivity is represented by the logarithmic *x*-axis. NP: nonpregnant mice, P: pregnant mice.

Focusing on CD4 positivity and the density of *γδ*TCR on *γδ*T cells, we discovered a lower intensity of *γδ*TCR expression on CD4+ *γδ*T cells independent of the tissue's origin (12.32 ± 2.58 vs. 20.5 ± 2.73 (*p* ≤ 0.01) in nonpregnant splenic, 16.18 ± 2.62 vs. 22.19 ± 2.53 (*p* ≤ 0.01) in pregnant splenic, 18.54 ± 4.19 vs. 48.44 ± 11.18 (*p* ≤ 0.05) in endometrial, and 11.59 ± 1.49 vs. 35.15 ± 4.47 (*p* ≤ 0.01) in decidual samples) (Figure 3(a)). Relating to CD8 positivity, a similar, significant alteration in *γδ*TCR expression was detected (15.3 ± 3.7 vs. 21.4 ± 2.4 (*p* ≤ 0.05) in nonpregnant splenic, 15.36 ± 1.9 vs. 43.8 (*p* ≤ 0.05) in endometrial, and 15.24 ± 1.93 vs. 29.78 (*p* ≤ 0.02) in decidual samples). However, no significant difference was detected in pregnant splenic samples (16.21 ± 1.97 vs. 19.95 ± 2.11) (Figure 3(b)).  Figure 3(c) shows the representative dotplot and histogram analyses of CD4 or CD8 and *γδ*TCR expression of lymphocytes.

### 3.3. CD107a and TIM-1, TIM-3, and CD160 Expression Profile of *γδ*T Cells Depending on the Isolated Tissue's Origin in Murine Pregnancy

**Figure 4:**
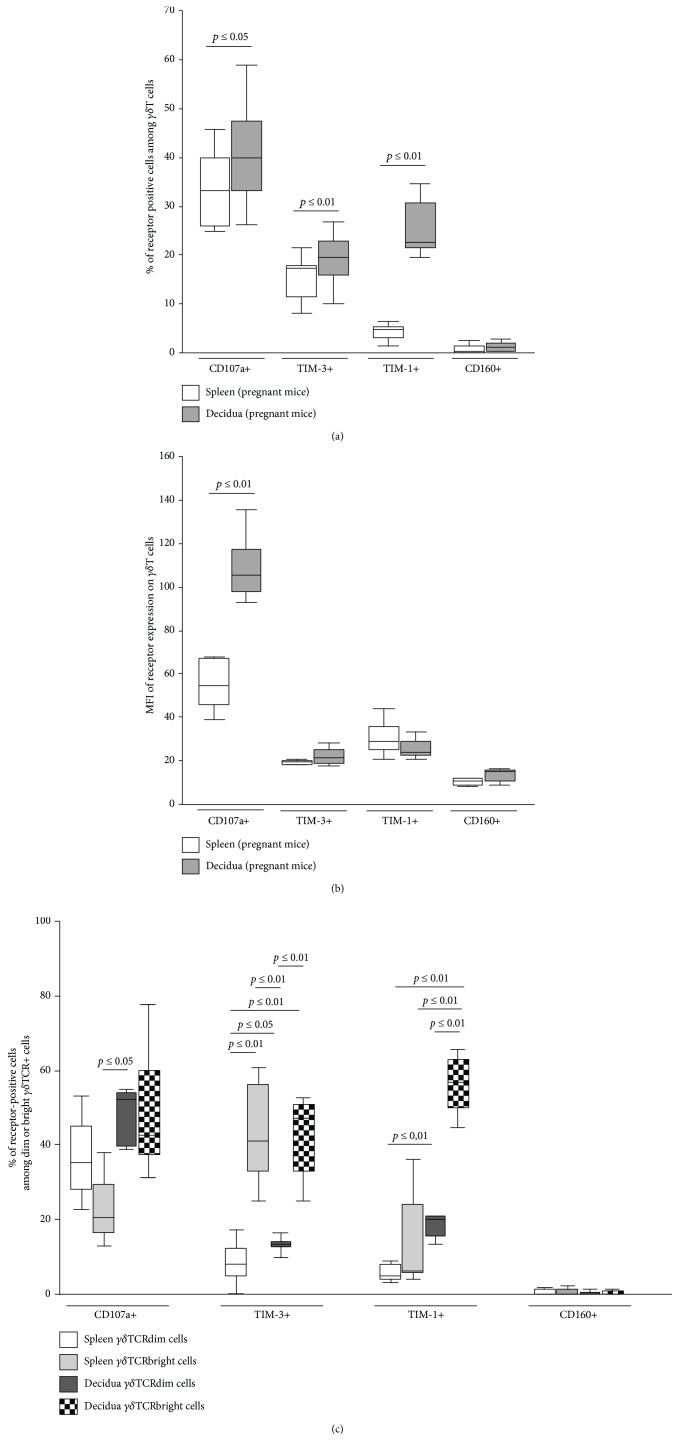
Receptor profile of *γδ*T cells in pregnant mice. (a) Ratio of CD107a, TIM-3, TIM-1, or CD160 receptor-positive cells among *γδ*T cells in pregnant mice. (b) Intensity of CD107a, TIM-3, TIM-1, or CD160 receptor expression on *γδ*T cells in pregnant mice. (c) Ratio of CD107a, TIM-3, TIM-1, or CD160 receptor-positive cells within *γδ*TCRdim and *γδ*TCRbright subsets in pregnant mice. The middle line of the boxplots represents the median, the lower box bound the first quartile, and the upper box bound the third quartile. The 95% confidence interval of the mean is represented by whiskers. Each boxplot represents results of at least seven independent experiments.

To get a clearer idea of the function of *γδ*T cells at the fetomaternal interface, we investigated their potential cytotoxicity by assessing their CD107a expression upon stimulation [36]. This marker of degranulation is significantly more often expressed by *γδ*T cells of the decidua than the spleen (40.75 ± 3.66 in the decidua vs. 33.65 ± 3.27 in the spleen, *p* ≤ 0.05). We also wanted to examine the cells for their immunoregulatory function. Thus, we analyzed the expression of TIM-3, TIM-1, and CD160 on *γδ*T cells. The rate of TIM-3-expressing cells among *γδ*T cells in the decidua is slightly but significantly higher than that in the spleen (19.22 ± 1.92 in the decidua vs. 15.36 ± 1.62 in the spleen, *p* ≤ 0.01). One-third of decidual *γδ*T cells show TIM-1 positivity, which is six times more often than the splenic *γδ*T cells do (28.45 ± 4.57 in the decidua vs. 4.25 ± 0.58 in the spleen, *p* ≤ 0.01). CD160 is rarely expressed on *γδ*T cells, and we did not find any significant alterations in the rate of positive cells for this receptor depending on the location of the cells (1.27 ± 0.38 in the decidua vs. 0.81 ± 0.35 in the spleen) (Figure 4(a)).

Upon investigating the expression level of functional markers, we detected a significantly higher intensity of CD107a expression (indicated by the higher MFI of CD107a) on decidual *γδ*T cells than on splenic *γδ*T cells (134.43 ± 25.81 in the decidua vs. 77.72 ± 23.32 in the spleen, *p* ≤ 0.01). Regarding the expression intensity of other functional markers, no significant difference was detected between the groups (TIM-3: 21.99 ± 1.36 in the decidua vs. 23.30 ± 5.41 in the spleen; TIM-1: 25.80 ± 2.00 in the decidua vs. 30.63 ± 2.77 in the spleen; and CD160: 15.03 ± 2.43 in the decidua vs. 10.40 ± 1.83 in the spleen) (Figure 4(b)).

Furthermore, the expression of functional markers is also connected to the intensity of *γδ*TCR expression on the respective cells. When gating the *γδ*TCRdim and *γδ*TCRbright subsets in pregnant mice, we found a significantly higher rate of CD107a+ cells among decidual *γδ*TCRdim lymphocytes compared to the splenic *γδ*TCRbright subset (48.0 ± 3.5 in decidual *γδ*TCRdim cells vs. 23.0 ± 5.3 in splenic *γδ*TCRbright cells, *p* ≤ 0.05), while there was no significant difference detected between the organ-related *γδ*TCRdim or *γδ*TCRbright subsets.

When investigating the ratio of TIM-3+ cells, we detected a significantly higher percentage of TIM-3+ cells among the *γδ*TCRbright subsets within each organ (spleen: 7.3 ± 2.9 of *γδ*TCRdim cells vs. 43.3 ± 6.9 of *γδ*TCRbright cells, *p* ≤ 0.01; and decidua: 13.5 ± 1.0 of *γδ*TCRdim cells vs. 42.3 ± 5.5 of *γδ*TCRbright cells, *p* ≤ 0.01). Furthermore, the ratio of TIM-3+ cells in the decidual *γδ*TCRdim subset was significantly higher than that in the splenic *γδ*TCRdim subset (*p* ≤ 0.05).

Interestingly, in the decidua, the rate of TIM-1+ cells was almost three times higher among the *γδ*TCRbright subset (56.0 ± 3.9) than among the *γδ*TCRdim one (18.3 ± 1.6, *p* ≤ 0.01). Although a similar tendency could be observed in the splenic samples, it did not reach the level of significance.

Finally, we could not detect any significant difference in the rate of CD160+ cells between the investigated groups (Figure 4(c)).

### 3.4. The Role of *γδ*T Cells in Specific Functional Phenotypes

**Figure 5:**
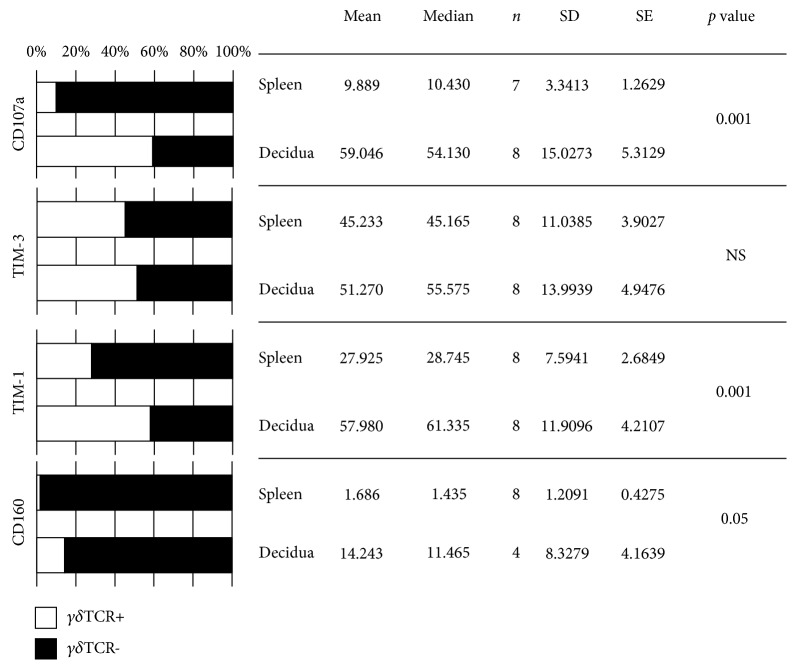
Gamma/delta T cells in various functional lymphocytic phenotypes. The mean rate of *γδ*TCR+ and *γδ*TCR− cells within CD107a+, TIM-3+, TIM-1+, and CD160+ lymphocytes in the spleen or decidua of pregnant mice shown as bars with related data. NS: not significant.

In this part of this study, we investigated the ratio of *γδ*T cells in different functional subsets of lymphocytes. The portion of *γδ*T cells among CD107a-expressing lymphocytes is six times greater in the decidua than in the spleen (59.05 ± 5.31 in the decidua vs. 9.89 ± 1.26 in the spleen, *p* ≤ 0.001). TIM-3 lymphocytes are one-half each *γδ*TCR+ or *γδ*TCR-, with no significant difference between the decidua and spleen (51.27 ± 4.95 in the decidua vs. 45.23 ± 3.90 in the spleen). However, one-half of the TIM-1+ lymphocytes are *γδ*TCR+ in the decidua, which are twice as many as in the spleen (57.98 ± 4.21 in the decidua vs. 27.93 ± 2.68 in the spleen, *p* ≤ 0.001). Although just a small percentage among the CD160+ lymphocytes show *γδ*TCR positivity, eight times more lymphocytes are *γδ*TCR+ in the decidua compared to the spleen (14.24 ± 4.16 in the decidua vs. 1.69 ± 0.43 in the spleen, *p* ≤ 0.05) (Figure 5).

## 4. Discussion

Gamma/delta T cells seem to play a part in the maintenance of a healthy pregnancy. Their effect appears to be primarily localized to the fetomaternal interface. In this study, we focused on the prevalence and the possible functions of *γδ*T cells in murine pregnancy.

In terms of CD4 expression, decidual *γδ*T cells are outstanding: approximately half of them are CD4+, which is twice as much as in the endometrium, pregnant spleen, or nonpregnant spleen. In contrast, CD8 expression is similar in all groups. Due to the design of the study, a possible double positivity could not be ruled out. Here, we demonstrate for the first time that CD4 and CD8 receptor coexpression with *γδ*TCR depends on the intensity of the *γδ*TCR expression. We were able to describe two distinct populations: a *γδ*TCRdim one, which was mainly CD4+ or CD8+ and a *γδ*TCRbright one, which was predominantly negative for CD4 and CD8. The presence of these *γδ*T subpopulations is particularly characteristic among resident *γδ*T cells. Hence, we assume that subsets within the *γδ*T cell population with altering functional characteristics should exist in the decidua. Those subsets might show similar variations like CD56dim and CD56bright NK cells. Our findings are in contrast with the results of Mincheva-Nilsson et al., who described human decidual *γδ*T cells as a CD4/CD8 double-negative population, although this result was based on a single human donor flow cytometric experiment [37]. Further investigations are necessary to clarify this contradiction. However, this difference could also be traced back to the discrepancy in the investigated species, although CD4+ *γδ*T cells have been described in other studies both in mice and in humans [38]. Murine CD4+ *γδ*T cells secrete high levels of Th2 cytokines [39]. Furthermore, after TGF*β* stimulation, naïve circulating *γδ*T cells mature into CD25+/FOXP3+ *γδ*T cells [40, 41]. Due to their Treg-like nature, it is presumable that those decidual *γδ*T cells express CD4, too. Additionally, it is possible that a Th2 subpopulation exists in addition to a Treg subpopulation within the *γδ*T cells at the fetomaternal interface.

Although *γδ*T cells with their significant cytokine production are able to form the local Th1-Th2 balance, which is an indispensable part of the immunotolerance towards the semiallogenic fetus, the precise regulation of this *γδ*T cell function at the fetomaternal interface is still not well understood. Here, we described that the prevalence of *γδ*T cells expressing TIM-3, commonly expressed by Th1 cells, is slightly higher in the decidua than in the spleen. It suggests that upon binding its ligand Gal-9, which is expressed in the placenta, TIM-3 could inhibit the Th1 function on *γδ*T cells [42]. Interestingly, the prevalence of TIM-1+ *γδ*T cells is six times higher in the decidua than in the spleen. TIM-1 is mainly found on Th2 cells and about half of the TIM-1-positive lymphocytes are *γδ*T cells in the decidua, which is twice as many as in the spleen. When investigating the expression of functional markers within the newly described *γδ*TCRbright and *γδ*TCRdim subsets, it appears that the above immune checkpoint molecules are mainly expressed on the *γδ*TCRbright subset. We assume that those cells could be regulated by TIM-3 and TIM-1, while the impact of these markers seems to be less pronounced in the *γδ*TCRdim subset, which might be rather controlled by CD4 or CD8. The fact that the rate of TIM-1+ cells in the decidual *γδ*TCRbright subset is so outstanding might be correlated to their activity at the fetomaternal interface, since TIM-1 is known to be upregulated upon activation [43]. These findings indicate a shift in the Th1-Th2 profile of *γδ*T cells towards the anti-inflammatory Th2 side in the decidua compared to the spleen. This is in line with the previously described Th2 bias in the decidua and the findings of McGrath et al., who proved that TIM-4, a natural ligand of TIM-1, promotes tolerance at the fetomaternal interface [44].

Approximately one-third of decidual *γδ*T cells express CD107a, and the density of its surface expression is significantly higher on decidual *γδ*T cells than on splenic *γδ*T cells. This phenomenon could be due to the character of the *γδ*TCRbright subset, which had significantly higher CD107a expression intensity than the *γδ*TCRdim one (data not shown). The cytotoxic activity of the decidual *γδ*TCRdim and *γδ*TCRbright cells could be regulated by CD8 or TIM-3 molecules, respectively. Among CD107a-expressing lymphocytes, over 50% are *γδ*T cells in the decidua, whereas in the spleen, only 10% of these lymphocytes are *γδ*T cells. Our results indicate that decidual *γδ*T cells might conserve their cytotoxic potential in the microenvironment of the fetomaternal interface, which could be useful in the protection against pathogens, as well as in the process of remodeling of the uterine tissue during placentation. In summary, it is easy to assume that diverse phenotype-related functions can be fulfilled by decidual *γδ*T cells.

The activity of each decidual *γδ*T cell subsets is largely determined by their coreceptors. CD160 plays a special role as a coreceptor, since both activation and inhibition can be mediated upon its binding. In CD4+ T cells, CD160 acts as an inhibitory coreceptor [24], whereas its signaling activates CD8+ T cells [45]. In our model, only a very small percentage of *γδ*T cells expressed CD160. Thus, we assume that CD160 plays a minor role in the regulation of *γδ*T cell function. Whether the activation upon CD160 binding influences *γδ*T cells at the fetomaternal interface or *γδ*T cells can upregulate CD160 under certain conditions at different time points during pregnancy is so far unknown.

## 5. Conclusions

Gamma/delta T cells have been suggested to promote tolerance towards the semiallogenic fetus in pregnancy. This is in line with our findings, and due to their TIM-1 positivity, decidual *γδ*T cells could be involved in the formation of local pregnancy-specific Th2 bias. Based on our results relating the different decidual *γδ*T cell subsets, we suppose that each phenotypic subpopulation, CD4-/CD8-positive *γδ*TCRdim or TIM-3-/TIM-1-positive *γδ*TCRbright, could correspond to a distinct function, which presumption needs further verification. In summary, beside cytotoxic potency, decidual *γδ*T cells appear to have an additional, pronounced regulatory capacity in the placental microenvironment by showing a tolerance promoting Th2-like phenotype. Further investigations are necessary to explore the exact functional consequences of our findings.

## Data Availability

The flow cytometric data used to support the findings of this study are available from the corresponding author upon request.
